# Individual Differences in Emotion Regulation, Childhood Trauma and Proneness to Shame and Guilt in Adolescence

**DOI:** 10.1371/journal.pone.0167299

**Published:** 2016-11-29

**Authors:** Aurora Szentágotai-Tătar, Andrei C. Miu

**Affiliations:** 1 Department of Clinical Psychology and Psychotherapy, Babeș-Bolyai University, Cluj-Napoca, Cluj, Romania; 2 Cognitive Neuroscience Laboratory, Department of Psychology, Babeș-Bolyai University, Cluj-Napoca, Cluj, Romania; Central Institute of Mental Health, GERMANY

## Abstract

Dispositional shame and guilt have been associated with psychopathology and an increasing number of studies have traced this relation back to adolescence. This developmental period is thought to be characterized by maturational changes in emotion regulation, which also play an important role in vulnerability to psychopathology, but little is known about the links between emotion regulation and dispositional shame and guilt. The current study investigated the relations between individual differences in the habitual use of a wide range of emotion regulation strategies and proneness to shame and guilt in a large sample of adolescents (N = 706), aged 13 to 17 years. History of childhood trauma was also assessed. Our results showed that emotion regulation independently explained about 20% of the variance of shame-proneness and guilt-proneness. Higher use of maladaptive (e.g., Self-Blaming, Catastrophizing) and lower use of adaptive (e.g., Refocus on Planning, Positive Reappraisal) emotion regulation strategies were positively associated with shame-proneness. In contrast, lower use of maladaptive (e.g., Catastrophizing, Blaming Others) and higher use of adaptive (e.g., Refocus on Planning, Positive Reappraisal) emotion regulation strategies were associated with guilt-proneness, independent of the influence of childhood trauma, which also explained a relatively minor portion of guilt-proneness. Although there were age differences (i.e., rumination was used more by older adolescents, and the influence of emotion regulation on depression and anxiety symptoms increased with age) and sex differences (i.e., girls reported higher use of Putting into Perspective and Other Blaming compared to boys) in emotion regulation, age and sex were not significantly associated with proneness to shame and guilt. The positive relations with maladaptive emotion regulation underscores the dysfunctional nature of shame-proneness. Future studies could use longitudinal measures to establish that emotion regulation drives dispositional shame and guilt, and also investigate whether emotion regulation optimization is able to normalize proneness to shame and guilt and reduce risk for psychopathology.

## Introduction

Self-conscious emotions are a special class of emotions that involve people's reactions to their own characteristics and behavior [1]. Shame and guilt are negatively valenced self-conscious emotions, typically experienced in situations of failure or in which behavioral standards are violated [1, 2]. Although they are elicited by similar types of situations, shame and guilt differ in terms of how individuals appraise transgressions or errors and in terms of the action tendencies they elicit [1, 3]. Shame usually involves negative evaluations of the global self, and is accompanied by a sense of inferiority and worthlessness, and the desire to escape or hide [1]. Guilt, on the other hand, involves the negative evaluation of a specific behavior, is characterized by remorse and regret over the bad thing done, and motivates reparative behavior [1].

Normative levels of shame and guilt are functional and serve social goals [2]. They have been linked to empathy towards others [4, 5], prosocial behavior [6–8], self-improvement motivation and behavior [9], and lower levels of aggression and antisocial behavior [10, 11]. However, when shame and guilt are disproportionate to the situation, and/or triggered very easily and experienced in a variety of contexts, becoming the dominant way of emotional responding, they can be problematic [2]. Studies on children and adolescents have linked shame-proneness to negative outcomes such as anxiety and depression [12–14], eating disorders, delinquent behavior and substance use (for review see [2, 15, 16]). Data regarding the association between guilt-proneness and psychological problems are less consistent [2]. They seem to indicate that guilt over specific behaviors is not associated with poor psychological adjustment [1, 17], and that guilt becomes maladaptive when it is fused with shame, when people develop a distorted sense of responsibility for events beyond their control, and when opportunities for reparation are blocked [1, 18, 19].

In order to be elicited, shame and guilt require self-awareness, the ability to form stable self-representations, to reflect on those representations, and to generate self-evaluations [20]. Being the product of complex cognitive operations, they emerge later in development than basic emotions, and are gradually shaped and refined through childhood and adolescence [10, 21]. So far, research on the development of self-conscious emotions has mainly focused on childhood [22, 23], and relatively little is known about their course across other life periods [24]. Existing data suggest that adolescence may be a particularly salient time in the development of shame-proneness and guilt-proneness. A previous study has shown that shame-proneness decreases from adolescence to middle adulthood [24]. However, another study reported that shame temporarily decreases in 10th grade adolescent girls and increases in 10th grade adolescent boys compared to 8th graders, but these changes are reversed in first year college students [25]. In which guilt-proneness is concerned, there seems to be a steady increase from adolescence to old age [24, 25]. Clearly, additional studies are needed in order to characterize age- and sex-related changes in shame-proneness and guilt-proneness in adolescence.

Several studies have also sought to understand the influence of childhood trauma on dispositional shame and guilt and found that neglect is associated with higher shame-proneness, but not guilt-proneness in children [26] and adults [19, 27]. Similarly, a recent longitudinal study has reported that harsh parenting in childhood is related to increased shame-proneness, but not guilt-proneness in adolescence [28]. Other childhood traumatic events such as parental conflict and sexual abuse were not associated with proneness to shame and guilt [28, 29]. Another recent study showed that shame-proneness may be increased in adolescents with a history of serious illness or injury [29]. Research focusing on situational shame and guilt has also documented their relation to childhood trauma. For instance, Alessandri and Lewis [30] found that maltreated children show higher levels of shame when they fail on a task, and Donatelli, Bybee, and Buka [12] found that adolescents whose mothers have a history of depression report more guilt over failing to meet maternal expectations. Overall, evidence on the impact of childhood trauma on shame and guilt in adolescence is heterogeneous, and this issue needs further clarification [17]. Crucially, studies on childhood trauma and shame and guilt need to control for traumatic intensity in order to ascertain that exposure to a childhood stressful event has a substantial negative impact on personality and life course [31], while also distinguishing between dispositional (i.e., proneness to shame and guilt) and domain or situation-specific shame and guilt.

Recent research suggests that the long-term influence of childhood trauma on shame-proneness and guilt-proneness in adolescence may involve other individual differences [28, 29]. One obvious candidate is emotion regulation, considering that it undergoes major maturational changes during adolescence (e.g., [32]), and plays a central role in emotional adaptation and risk for psychopathology (e.g., [33]). Adolescence may be characterized by changes both in the habitual use of emotion regulation strategies and the efficiency of these strategies, as reflected in their relations with emotional difficulties [34]. To our knowledge, there is only limited evidence regarding the links between emotion regulation and proneness to shame and guilt. For example, a recent study [35] has found that higher use of suppression (i.e., inhibiting emotional expressions) is associated with increased shame-proneness, whereas higher use of reappraisal (i.e., changing the meaning of a situation) is associated with increased guilt-proneness in adolescence. These results suggest that the preference for maladaptive emotion regulation strategies, which are less efficient in reducing negative affect (e.g., suppression), may be related to shame-proneness, whereas preference for adaptive, more efficient strategies (e.g., reappraisal) may be related to guilt-proneness. Indeed, emotion regulation efficiency (i.e., impulse and anger control; tendency to down-regulate negative affect and up-regulate positive affect; appropriateness of emotional responses relative to the situation) was found to be negatively correlated with shame-proneness, and positively correlated with guilt-proneness [35, 36]. However, adolescents use many emotion regulation strategies when facing negative events (e.g., [37]) and the analysis of the relations between habitual emotion regulation and proneness to shame and guilt should be extended. Research shows that other dimensions of negative affect (e.g., depression, anxiety) are positively associated with higher use of emotion regulation strategies such as rumination, self-blame and catastrophizing, and negatively associated with higher use of strategies such as positive refocusing and positive reappraisal [34, 37], but the links between these strategies and proneness to shame and guilt have not been examined until now.

The present study investigated the independent contributions of age, sex, childhood trauma and individual differences in emotion regulation to shame-proneness and guilt-proneness in a large community sample of 13 to 17-year-old adolescents. Following recommendations in the field [28, 38], we used derivate measures of shame-proneness and guilt-proneness, which control for the interrelations between these dispositional dimensions. In addition, we focused on childhood negative events that were perceived as highly traumatic, in order to reduce heterogeneity in stressor intensity [31]. Moreover, we linked shame-proneness and guilt-proneness with depression and anxiety symptoms, to highlight their contribution to vulnerability to psychopathology [28]. Finally, individual differences in emotion regulation were assessed using a multidimensional scale that captures the habitual use of a wide range of adaptive and maladaptive cognitive emotion regulation strategies [37].

## Materials and Methods

### Participants

The present sample included 706 adolescents (43.5% girls), aged between 13 and 17 years (*M* = 15.63; *SD* = 1.20). They were recruited through advertisements from several regions of Romania. Romanian was the first language of all participants. Written parental consent and participant assent were obtained from all participants prior to the study. Participants filled in all questionnaires in one session. This study was approved by the Ethics Committee of Babeș-Bolyai University, and was conducted in accordance with the ethical standards laid down in the 1946 Declaration of Helsinki and its later amendments.

### Measures

Childhood trauma was investigated using a Romanian translation of the Childhood Traumatic Events Scale [31], which was successfully used in previous studies (e.g., [39]). This self-report measure assesses several types of traumatic events experienced before age 17 (or until the present in participants of younger ages): (1) death of a very close friend or family member; (2) major upheaval between parents, such as separation or divorce; (3) sexual abuse, such as rape or molestation; (4) violent events, such as physical abuse, mugging or assault; and (5) severe illness or injury. Participants are asked to report whether they have experienced each type of stressful event and if they have, they also rate its severity on a 7-point scale, where 1 stands for "not at all traumatic", 4 for "somewhat traumatic", and 7 for "extremely traumatic". Following Pennebaker and Susman [31], only events that received scores above 6 on the traumatic intensity scale were considered in this study.

The Romanian version [40] of the Cognitive Emotion Regulation Questionnaire (CERQ) [37] was used to assess individual differences in emotion regulation. CERQ is a self-report measure of the habitual frequency of using the following emotion regulation strategies when confronted with stressful events: (1) Self-Blaming (i.e., putting the blame for the event on yourself) (Cronbach's alpha = 0.68 in this sample); (2) Acceptance (i.e., coming to terms with the event) (Cronbach's alpha = 0.65 in this sample); (3) Rumination (i.e., repetitively thinking about the event and related emotions) (Cronbach's alpha = 0.75 in this sample); (4) Positive Refocusing (i.e., thinking about positive issues instead of the event) (Cronbach's alpha = 0.71 in this sample); (5) Refocus on Planning (i.e., addressing the steps necessary to handle the situation) (Cronbach's alpha = 0.64 in this sample); (6) Positive Reappraisal (i.e., giving the event some sort of positive meaning) (Cronbach's alpha = 0.74 in this sample); (7) Putting into Perspective (i.e., playing down the seriousness of the event) (Cronbach's alpha = 0.72 in this sample); (8) Catastrophizing (i.e., thinking about how bad the event is) (Cronbach's alpha = 0.72 in this sample); and (9) Blaming Others (i.e., putting the blame for the event on the situation or other people) (Cronbach's alpha = 0.68 in this sample). Each subscale consists of four items, which are rated from 1 (almost never) to 5 (almost always). A subscale score is obtained by adding up the four items, and subscale scores range from 4 to 20. Reliability coefficients obtained in this sample are similar to those reported by Garnefski, Kraaij, and Spinhoven [37], and acceptable considering the small number of items in each subscale.

Shame-proneness and guilt-proneness were assessed using the Test of Self-Conscious Affect for Adolescents (TOSCA-A) [41]. We used a Romanian translation that has been employed in previous studies (e.g., [29]) and shows reliability coefficients (see below) similar to those reported for the original scale [41]. TOSCA-A consists of 15 scenarios, 10 negative and 5 positive, yielding indices of shame-proneness (Cronbach's alpha = 0.79 in this sample) and guilt-proneness (Cronbach's alpha = 0.84 in this sample). Each scenario (e.g., "You and your friend are talking in class, and you get in trouble") is followed by a list of possible responses (e.g., "I would feel like everyone in the class was looking at me and they were about to laugh" for shame; or "I would think: I should know better. I deserve to get in trouble" for guilt). Participants rate the likelihood of each response on a scale ranging from 1 (not at all likely) to 5 (very likely).

The Romanian version [42] of the Depression Anxiety Stress Scales (DASS) [43] was used to assess depression symptoms (e.g., hopelessness, lack of interest) and anxiety symptoms (e.g., subjective apprehension, autonomic arousal). Each of these subscales includes 7 items, which are appropriate for adolescents [44] and show good sensitivity to clinical levels of emotional symptoms [45]. In this sample, Cronbach's alpha was 0.81 for the depression subscale, and 0.74 for the anxiety subscale.

### Statistical Analyses

The main objective of this study was to identify the influence of childhood trauma and emotion regulation on shame-proneness and guilt-proneness in adolescence. Listwise deletion was used to handle missing data, which represented 1.39% of all cases. Outlier analysis identified two extreme values, but the results did not change after excluding them; therefore, they were kept in the analyses. Several preliminary analyses were first run in order to clarify four important issues.

First, the distribution of childhood trauma in the present sample was described. Considering that we focused on events rated as "extremely traumatic", which may be rare in the typical sample, we recoded this variable by creating a dummy that compared participants with and without childhood trauma.

Second, we examined the correlations between age and the habitual use of the emotion regulation strategies assessed by CERQ, and between the latter and DASS depression and anxiety symptoms. Sex differences in emotion regulation and emotional symptoms were also investigated using Student t-tests for independent samples.

Third, the interrelations between TOSCA-A shame and guilt scores were investigated. Given that TOSCA-A is designed so that responders can endorse both shame and guilt responses, raw scores are often closely correlated and standardized residual scores are recommended as more valid measures of shame-proneness (while controlling for guilt-proneness) and guilt-proneness (while controlling for shame proneness) [28, 38]. In order to describe differential links with emotional dysfunctions, we examined the correlations between shame-proneness and guilt-proneness, on the one hand, and DASS depression and anxiety scores, on the other hand.

Fourth, given the potential conceptual overlap between emotional dispositions (e.g., shame-proneness) and some of the emotion regulation strategies (e.g., CERQ Self-Blaming), we also examined the magnitude of the correlations between these measures. Large correlations (i.e., > 0.50) were considered indicative of substantial conceptual overlap [46].

The main analyses involved hierarchical multiple regression [47], in which shame-proneness and guilt-proneness were separately regressed on age and sex (entered in Step 1), history of childhood trauma (Step 2), and individual differences in emotion regulation (Step 3). This allowed us to characterize the independent contributions of these categories of predictors.

All analyses were run in SPSS. Where the case, the threshold of statistical significance was corrected for multiple testing, using the Bonferroni method (e.g., [48]).

## Results

### Childhood Trauma Reports

Most adolescents (85%) reported no history of childhood trauma. The rest of the sample reported one (13.2%), two (0.8%), three (0.7%) or four (0.3%) childhood traumatic events. None of the participants reported five traumatic events. The frequency of specific traumatic events was as follows: death of a very close friend or family member (11.8%); major upheaval between parents (2.7%); severe illness or injury (1.8%); violent events (1.1%); and sexual abuse (0.7%). Considering the low number of reports of each type of childhood trauma, all subsequent analyses focused on the total number of childhood traumatic events. More specifically, given that very few participants (i.e., 1.8%) reported multiple traumatic events, we compared between adolescents with a history of childhood trauma (i.e., one or more traumatic events) and adolescents without such a history (i.e., no traumatic event).

### Age, Sex and Emotional Symptoms

First, we investigated the relations between age and the habitual use of emotion regulation strategies. Age was positively associated with CERQ Rumination (two-tailed *r* = 0.13, *p* = 0.001). There were no other significant correlations between age and CERQ emotion regulation scores. Moreover, the correlations between age and DASS depression (two-tailed *r* = 0.06, *p* = 0.090) and anxiety scores (two-tailed *r* = 0.03, *p* = 0.313) were not significant.

Table 1 shows the correlations between emotion regulation and emotional symptoms. CERQ Self-Blaming, Acceptance, Rumination, Catastrophizing and Blaming Others scores correlated positively with both DASS depression and DASS anxiety scores. In addition, CERQ Putting into Perspective was positively associated with DASS anxiety. In contrast, CERQ Positive Refocusing, Refocus on Planning and Positive Reappraisal scores correlated negatively with DASS depression scores.

**Table 1:** Correlations between emotion regulation and depression and anxiety symptoms.

Questionnaire		DASS	
		Depression	Anxiety
CERQ	Self-blaming	0.33**	0.27**
	Acceptance	0.10**	0.17**
	Rumination	0.32**	0.30**
	Positive Refocusing	-0.08*	-0.04
	Refocus on Planning	-0.08*	0.05
	Positive Reappraisal	-0.16**	-0.03
	Putting into Perspective	-0.03	0.11**
	Catastrophizing	0.41**	0.34**
	Blaming Others	0.20**	0.17**

*Note*: Values in cells are two-tailed Pearson r coefficients. Abbreviations: CERQ, Cognitive Emotion Regulation Questionnaire; DASS, Depression Anxiety Stress Scales.

* p < 0.05;

** p < 0.01.

Using Student t-tests for independent samples, we also checked for sex differences in emotion regulation and emotional symptoms. Girls reported significantly higher levels of CERQ Putting into Perspective (*t*[644] = 2.25, *p* = 0.024) and significantly lower levels of CERQ Other Blaming (*t*[644] = -3.01, *p* = 0.003) in comparison to boys. There were no other significant sex differences in the other CERQ emotion regulation scores or in DASS depression and anxiety scores (all *p*s> 0.105).

### Shame-Proneness, Guilt-Proneness and Emotional Symptoms

There was a significant association between TOSCA-A shame-proneness and guilt-proneness scores (*r* = 0.40, *p*< 0.001). In order to control for this relation, standardized residual scores of "guilt-free" shame-proneness and "shame-free" guilt-proneness were used in all subsequent analyses.

Shame-proneness residual scores positively correlated with DASS depression (two-tailed *r* = 0.41, *p*< 0.001) and DASS anxiety scores (two-tailed *r* = 0.31, *p*< 0.001). Guilt-proneness residual scores negatively correlated with DASS depression (two-tailed *r* = -0.18, *p*< 0.001), but they did not correlate with DASS anxiety scores (two-tailed *r* = 0.01, *p* = 0.920).

The correlations between CERQ scores and TOSCA-A shame and guilt scores were small or moderate (all *r*s< 0.37) and therefore, substantial conceptual overlap between emotion regulation, on the one hand, and shame-proneness and guilt-proneness, on the other hand, was considered unlikely.

### Predictors of Shame-Proneness and Guilt-Proneness

Two separate regression analyses focused on shame-proneness residual scores and guilt-proneness residual scores as outcomes, and age and sex (Step 1), childhood trauma (Step 2) and CERQ emotion regulation (Step 3) as predictors. Sex (boys = 0; girls = 1) and childhood trauma (no traumatic event = 0; one or more traumatic events = 1) were dummy coded. Tolerance (all ≤ 0.99) and variance inflation factor (all ≤ 2.21) coefficients did not indicate multicollinearity. Given that two regression models were tested, a Bonferroni-corrected threshold of statistical significance (*p*/2 = 0.025) was adopted for these analyses.

The model in which shame-proneness was used as outcome was not significant in Step 1 (*F*[2, 637] = 1.34, *p* = 0.262) and Step 2 (*F*[3, 636] = 0.90, *p* = 0.439), which indicated that neither age and sex, nor the history of childhood trauma were significantly related to shame-proneness (Table 2). The model became significant (*F*[12, 627] = 15.60, *p*< 0.001) in Step 3, after CERQ emotion regulation scores were added, and accounted for an additional 22.57% of shame-proneness (*F*-change[9, 627] = 20.41, *p*< 0.001). As shown in Table 2, CERQ Self-Blaming, Positive Refocusing and Catastrophizing scores were significant positive predictors of shame-proneness, whereas CERQ Refocus on Planning and Positive Reappraisal scores were negative predictors of shame-proneness.

**Table 2:** Coefficients from the multiple regression in which shame-proneness was regressed on age and sex, childhood trauma and individual differences in emotion regulation.

Step and variable		B	SE B	95% CI	Beta	R^2^
Step 1	Age	0.05	0.03	-0.13, 0.07	0.05	0.004
	Sex (boys = 0; girls = 1)	0.04	0.07	-0.01, 0.11	0.02	
Step 2	Childhood trauma (no trauma = 0; one or more trauma = 1)	0.01	0.11	-0.20, 0.24	0.01	0.004
Step 3	CERQ Self-blaming	0.08	0.01	0.04, 0.11	0.21**	0.229**
	CERQ Acceptance	-0.02	0.01	-0.04, 0	-0.06	
	CERQ Rumination	0.01	0.01	-0.01, 0.04	0.05	
	CERQ Positive Refocusing	0.03	0.01	0.01, 0.05	0.10*	
	CERQ Refocus on Planning	-0.04	0.01	-0.07, -0.01	-0.13*	
	CERQ Positive Reappraisal	-0.05	0.01	-0.08, -0.02	-0.19**	
	CERQ Putting into Perspective	0.02	0.01	0, 0.04	0.08	
	CERQ Catastrophizing	0.07	0.01	0.04, 0.09	0.22**	
	CERQ Blaming Others	0.03	0.01	0, 0.06	0.07	

*Note*: B, unstandardized regression coefficient; Beta, standardized regression coefficient; CI, confidence interval; SE, standard error. Abbreviations: CERQ, Cognitive Emotion Regulation Questionnaire.

* p < 0.025;

** p < 0.001.

The model in which guilt-proneness was used as outcome was not significant in Step 1 (*F*[2, 637] = 3.18, *p* = 0.042). Neither age, nor sex was significantly related to guilt-proneness (Table 3). The model became significant (*F*[3, 636] = 5.56, *p* = 0.001) in Step 2, after adding the history of childhood trauma as predictor, and accounted for an additional 1.57% of the variance of guilt proneness (*F*-change[1, 636] = 10.22, *p* = 0.001). The history of childhood trauma was a significant positive predictor of guilt-proneness. The model remained significant (*F*[12, 627] = 14.59, *p*< 0.001) in Step 3, after entering CERQ emotion regulation scores as predictors, and accounted for an additional 19.27% of the variance of guilt-proneness (*F*-change[9, 627] = 17.17, *p*< 0.001). CERQ Refocus on Planning and Positive Reappraisal scores were significant positive predictors of guilt-proneness, and CERQ Positive Refocusing, Catastrophizing and Blaming Others scores were negative predictors of guilt-proneness.

**Table 3:** Coefficients from the multiple regression in which guilt-proneness was regressed on age and sex, childhood trauma and individual differences in emotion regulation.

Step and variable		B	SE B	95% CI	Beta	R^2^
Step 1	Age	-0.06	0.03		-0.08	0.009
	Sex (boys = 0; girls = 1)	0.12	0.07		0.06	
Step 2	Childhood trauma (no trauma = 0; one or more trauma = 1)	0.35	0.11		0.12**	0.025*
Step 3	CERQ Self-blaming	0.01	0.01		0.01	0.218**
	CERQ Acceptance	0.02	0.01		0.06	
	CERQ Rumination	0.02	0.01		0.07	
	CERQ Positive Refocusing	-0.03	0.01		-0.11*	
	CERQ Refocus on Planning	0.04	0.01		0.13*	
	CERQ Positive Reappraisal	0.06	0.01		0.21**	
	CERQ Putting into Perspective	0.02	0.01		0.08	
	CERQ Catastrophizing	-0.03	0.01		-0.10*	
	CERQ Blaming Others	-0.06	0.01		-0.17**	

*Note*: B, unstandardized regression coefficient; Beta, standardized regression coefficient; CI, confidence interval; SE, standard error. Abbreviations: CERQ, Cognitive Emotion Regulation Questionnaire.

* p < 0.025;

** p < 0.001.

## Discussion

The present results show that individual differences in emotion regulation are related to shame-proneness and guilt-proneness in adolescents, independent of the influence of age, sex and childhood trauma. Childhood trauma was related to guilt-proneness, but it explained only a relatively minor portion of its variance compared to emotion regulation. This study also indicates that the habitual use of rumination increases with age and described positive and negative associations between emotion regulation strategies and emotional symptoms in adolescence.

Individual differences in the habitual use of emotion regulation strategies explained around 20% of both shame-proneness and guilt-proneness. Remarkably, largely the same emotion regulation strategies contributed to both emotional dispositions, but with opposing roles. That is, we found that the more often one thinks about positive issues instead of confronting negative events (i.e., Positive Refocusing) and thinks about how bad negative events are (i.e., Catastrophizing), the higher s/he is on shame-proneness and the lower on guilt-proneness. In addition, the habitual use of putting the blame on oneself for negative events (i.e., Self-Blaming) is associated, as one would expect, with higher shame-proneness, whereas putting the blame on the situation or on other people (i.e., Blaming Others) is related to lower guilt-proneness. Extending the contrast, tendencies to confront negative events by taking the necessary steps to handle the situation (i.e., Refocus on Planning) and to look for a positive meaning of negative events (i.e., Positive Reappraisal) are linked with lower shame-proneness, and higher guilt-proneness in this study. Overall, the present results indicate that maladaptive emotion regulation strategies, such as Catastrophizing, Self-Blaming and Blaming Others, which have been associated with depression and anxiety symptoms in previous studies (e.g., [49, 50]) as well as in the present sample, are also related to shame-proneness, whereas low levels of these strategies are associated with guilt-proneness. In contrast, adaptive emotion regulation strategies such as Positive Reappraisal and Refocus on Planning, which are known to mitigate against symptoms of depression and anxiety (see [49] and this study), worry and fearfulness [50] are associated with reduced shame-proneness and higher guilt-proneness. Positive Refocusing is an exception to this pattern considering that, while it is viewed as an adaptive emotion regulation strategy based on its negative relation to depression symptoms (see [50] and this study), it has been associated with enhanced shame-proneness and reduced guilt-proneness in the present study. This relation may seem less surprising if we consider that both Positive Refocusing and shame involve disengagement from unpleasant situations, one by distraction with the help of positive thoughts about unrelated matters [37] and the latter, by an intrinsic desire to escape or hide in negative situations [1]. Importantly, given the correlational and cross-sectional nature of the present study, the direction of the relations between emotion regulation and proneness to shame and guilt cannot be identified. While the influence of emotion regulation on dispositional shame and guilt is more plausible considering evidence from prospective studies (e.g., [51]), which showed that emotion regulation predicts subsequent emotional adjustment and not the other way around, this study cannot rule out alternative models in which dispositional shame and guilt drive habitual emotion regulation or they influence each other.

The present results also show that guilt-proneness is increased in adolescents with a history of childhood trauma. Previous studies have reported that neglect [26], harsh parenting [28] and severe illness or injury [29] are associated with enhanced shame-proneness, but not guilt proneness. Our findings may thus seem at odds with this literature, but we argue that the discrepancy rests in methodological differences. The present study assessed a variety of childhood negative events, most of which were not investigated in previous research [26, 28]. We employed the same measure in one of our previous studies [29], but the analysis in that study did not control for traumatic intensity and therefore, a whole range of childhood negative events, from mild to traumatic, were included. In order to limit the heterogeneity of childhood stressors, the present study focused on traumatic events that were perceived by participants as having had a substantial impact on their personality and life course. As expected, only a minority of adolescents (i.e., 15%) reported such trauma, and we found that they had higher levels of guilt-proneness. The association between childhood trauma and guilt-proneness echoes previous observations that adolescents with depressive mothers tend to feel guiltier over failing to meet maternal expectations, compared to adolescents with non-depressive mothers [12]. Considering that the available literature on this topic includes only a handful of studies, future research should systematically describe the relations between different aspects of childhood trauma (e.g., type, severity, chronicity, age of exposure) and proneness to shame guilt.

We found no evidence for an association between age and sex, and dispositional shame and guilt in adolescents. A previous longitudinal study [24] showed that shame-proneness decreased and guilt-proneness increased from adolescence onward, with the former reaching a minimum around age 50, and the latter reaching a plateau around age 70. Therefore, age-related changes in shame-proneness and guilt-proneness may start in adolescence, but they extend into adulthood and this might explain why we found no association between age and these emotional dispositions in adolescents between ages 13 and 17. In which sex is concerned, a recent meta-analysis [52] has suggested that sex differences in shame and guilt are small, and this may account for the failure to detect such differences in the present study.

An important assumption of this study was that adolescence is marked by changes in emotion regulation [32], with a potential impact on the development of shame-proneness and guilt-proneness (e.g., [24]). Taking advantage of the large sample of adolescents included in the study, we were able to show that the habitual use of Rumination increases with age. Our results also illustrate the maladaptive role of emotion regulation strategies such as Self-Blaming, Rumination, Catastrophizing and Blaming Others, positively associated with depression and anxiety symptoms, and the adaptive role of strategies such as Positive Refocusing, Refocus on Planning and Positive Reappraisal, negatively associated with depression. The positive association between Acceptance and emotional symptoms may be explained by its specific efficiency in situations of uncontrollable stress (e.g., [53]). Putting into Perspective was also positively associated with anxiety, which underscores the potential negative consequences of playing down the seriousness of stressful events.

As expected [28, 38], raw scores of shame-proneness and guilt-proneness correlated, and therefore, derivate measures (i.e., standardized residual scores) were used to control for this interrelation. Using these indices, which may have improved construct validity [28], we found that shame-proneness was positively associated with depression and anxiety symptoms and guilt-proneness was negatively associated with depression symptoms. This underscores the dysfunctional nature of shame-proneness, which has been consistently linked to psychopathology [1]. The relation between guilt-proneness and psychopathology is less straightforward [1]. However, when guilt-proneness pervades emotional responses, it can also become problematic and contribute to emotional dysfunction [2]. This study was not designed to identify moderators in the relation between guilt-proneness and mental health, mainly because it did not employ a comprehensive psychiatric assessment, but this issue is an important challenge for future research.

Our results suggest that emotion regulation may be targeted in interventions aiming to reduce shame-proneness and prevent associated dysfunctions. Previous studies have shown that neglected children [26] and those exposed to harsh parenting [28] may be prone to exaggerated levels of dispositional shame and subsequent depression. By assessing ways of reducing the use of maladaptive emotion regulation strategies such as Self-Blaming and Catastrophizing, and enhancing the use of adaptive strategies such as Refocus on Planning and Positive Reappraisal, future research could examine the social and emotional benefits of reducing shame-proneness in children and adolescents.

The major limitation of this study is related to its correlational and cross-sectional nature, which does not allow us to draw a conclusion on the direction of the relations between habitual emotion regulation and dispositional shame and guilt. Future longitudinal studies and interventions targeting emotion regulation (e.g., promoting the use of adaptive strategies and discouraging the use of maladaptive strategies) will be able to characterize the relations between emotion regulation and proneness to shame and guilt. Another limitation of the present study is that it relied exclusively on self-report assessments of childhood trauma, emotion regulation, shame-proneness and guilt-proneness, and these measures may be susceptible to social and memory biases. For instance, childhood maltreatment may be underreported in questionnaires [54], and self-deceptive enhancement may inflate the self-report of socially desirable emotion regulation strategies such as reappraisal [55]. Therefore, future studies could use interview-based measures of childhood trauma (e.g., [54]) and cognitive tasks that directly assess the efficiency of emotion regulation strategies (e.g., [56]). In addition, it could be useful to differentiate between emotional and behavioral aspects of shame and guilt (e.g., [4]), and employ candidate biomarkers of these emotional dispositions (e.g., [57]).

In conclusion, these results describe the relations between individual differences in emotion regulation and dispositional shame and guilt in adolescence. In light of its positive association with the habitual use of maladaptive emotion regulation strategies and emotional symptoms, shame-proneness appears to be more dysfunctional than guilt proneness. Therefore, future research should seek ways of reducing shame-proneness in adolescence and we suggest that the optimization of emotion regulation might offer a promising mean of achieving this goal.
