# Correction: Individual Differences in Emotion Regulation, Childhood Trauma and Proneness to Shame and Guilt in Adolescence

**DOI:** 10.1371/journal.pone.0171151

**Published:** 2017-01-25

**Authors:** Aurora Szentágotai-Tătar, Andrei C. Miu

There are errors in the eighth and ninth sentences of the Abstract. The correct sentences are: Although there were age differences (i.e., rumination was used more by older adolescents) and sex differences (i.e., girls reported higher use of Putting into Perspective and lower use of Other Blaming compared to boys) in emotion regulation, age and sex were not significantly associated with proneness to shame and guilt. The positive relations with maladaptive emotion regulation underscore the dysfunctional nature of shame-proneness.
